# A Qualitative Study Exploring the Experiences of Carers of Service Users With Complex Mental Health Needs

**DOI:** 10.1192/bjo.2022.240

**Published:** 2022-06-20

**Authors:** Pooja Saini, Hana Roks, Laura Sambrook, Anna Balmer, Jason McIntyre, Antony Martin, Jackie Tait, Peter Ashley-Mudie, Amrith Shetty, Rajan Nathan

**Affiliations:** 1 Liverpool John Moores University, Liverpool, United Kingdom; 2 QC Medica LLP, York, United Kingdom; 3 Cheshire and Wirral Partnership NHS Foundation Trust, Chester, United Kingdom

## Abstract

**Aims:**

Carers of individuals presenting with complex behavioural and mental health needs report that service users may not receive the provision of care they require, particularly when presenting following suicide attempts and self-harm. Carers are an integral part of the care system and often feel ignored and marginalised by services; there is a lack of involvement of carers and paucity of their views of support needs to be explored. The aim of the study is to understand carers’ experiences of caring for service users with complex mental health needs who self-harm and/or attempt suicide, and the support received from mental health care services.

**Methods:**

Ten carers of service users with complex mental health needs were interviewed about their views on the psychiatric admission, treatment and discharge process for the people they were caring for. Data were gathered during semi-structured, one-to-one interviews remotely over the phone or online platforms. Interviews were audio-recoded and transcribed verbatim. A transcript-based conceptual analysis was conducted to identify and explore emerging themes.

**Results:**

Carers identified both positive and negative aspects of the psychiatric admission and care within community settings. The following key themes emerged from the interviews: lack of control and information from mental health services, the importance of support from staff, or conversely its absence; concerns about service users’ vulnerability, negative staff attitudes and opportunities for involvement; negative experiences of generic psychiatric settings; positive experiences were encountered when there were supportive and caring staff, good information sharing and satisfactory discharge arrangements.

**Conclusion:**

Important areas for service improvements are highlighted. Recommendations included: the need for support; information about suicidal behaviour and advice on managing further incidents at home; more support in coping with regular and escalating self-harming and suicidal behaviours, particularly, severe consequences of staff safeguarding errors and inappropriate discharge, and the importance of supportive and adept staff. These findings identify the need for tailored support for carers regarding the management of self-harm and suicidal behaviours in the community.

